# Two new species of *Cladosporium* from leaf spots of *Parispolyphylla* in north-western Yunnan Province, China

**DOI:** 10.3897/BDJ.9.e77224

**Published:** 2021-12-23

**Authors:** Yue-Xin Xu, Hong-Wei Shen, Dan-Feng Bao, Zong-Long Luo, Hong-Yan Su, Yu-E Hao

**Affiliations:** 1 College of Public Health, University of South China, Hengyang, China College of Public Health, University of South China Hengyang China; 2 College of Agriculture and Biological Sciences, Dali University, Dali, China College of Agriculture and Biological Sciences, Dali University Dali China; 3 Center of Excellence in Fungal Research, Mae Fah Luang University, Chiang Rai, Thailand Center of Excellence in Fungal Research, Mae Fah Luang University Chiang Rai Thailand; 4 School of Science, Mae Fah Luang University, Chiang Rai, Thailand School of Science, Mae Fah Luang University Chiang Rai Thailand; 5 Department of Entomology and Plant Pathology, Faculty of Agriculture, Chiang Mai University, Chiang Mai, Thailand Department of Entomology and Plant Pathology, Faculty of Agriculture, Chiang Mai University Chiang Mai Thailand

**Keywords:** asexual morph, *Cladosporium*, hyphomycetes, phylogeny, taxonomy

## Abstract

**Background:**

During the survey of pathogenic fungi on medicinal plant leaves in Yunnan Province, China, two *Cladosporium*-like taxa were isolated from leaf spots of *Parispolyphylla*. Based on morphological characteristics and phylogenetic analysis of combined ITS, TEF1-α and ACT genes, two new species were discovered.

**New information:**

Two new species *Cladosporiumyunnanensis* and *C.paris* are introduced, the detailed descriptions and illustrations are provided. Morphology of the two new species is compared with other related *Cladosporium* species. This study widens the host diversity of the genus *Cladosporium*.

## Introduction

*Cladosporium* is one of the largest and most heterogeneous genera of hyphomycetous fungi (Dugan et al. 2004). It was initially described by Persoon (1794) from rotten wood as *Dematiumherbarum* Pers., which was later synonymised by Link (1816) as *Cladosporiumherbarum* (Pers.: Fr.). *Cladosporium* is currently only known as the asexual morph, which is characterised by erect, straight or geniculate conidiophores, abundant branched acropetal chains of smooth to roughened dry conidia produced from mono- or polyblastic conidiogenous cells, the coronate structure of conidiogenous loci and conidial hila, consisting of a central convex dome surrounded by a raised periclinal rim (David 1997, Crous et al. 2007).

To clarify the relationship of species in the complex *Cladosporium*, subsequent researchers have been constantly revising this genus and the use of molecular analysis is necessary as well as morphological characters (David 1997, Dugan et al. 2004, Heuchert et al. 2005, Schubert 2005, Schubert et al. 2007, Crous et al. 2007, Sandoval-Denis et al. 2016, Bezerra et al. 2017, Bezerra et al. 2017, Abdollahzadeh et al. 2020). Some phylogenetic studies have proposed a multi-locus sequence analysis approach to clarify species diversity within the genus with internal spacers of the rDNA genes (ITS), translation elongation factor 1-α (TEF1-α) and actin (ACT) (Bensch et al. 2012, Bensch et al. 2015, Bensch et al. 2018, Tibpromma et al. 2019, Iturrieta-González et al. 2021, Zimowska et al. 2021). Based on the phylogenetic analyses and morphological features, about 237 species have been accepted within the genus, which are split into three species complexes, *Cladosporiumherbaum* (Schubert et al. 2007), *C.sphaerospermum* (Zalar et al. 2007, Dugan et al. 2008) and *C.cladosporioides* (Bensch et al. 2010).

The species of *Cladosporium* are able to colonise a wide range of substrates and can be isolated in any natural or anthropogenically-affected environment (Flannigan et al. 2002, Bensch et al. 2010, Bensch et al. 2012, Bensch et al. 2018, Sandoval-Denis et al. 2015, Temperini et al. 2018, Chung et al. 2019). They are well known as plant pathogens, which may occur on leaves, stems and fruits on different plants, for example, *Asparagaceae*, *Asteraceae*, *Fabaceae*, *Myrtaceae*, *Orchidaceae* and *Poaceae* (Schubert 2005, Bensch et al. 2012, Bensch et al. 2015, Marin-Felix et al. 2017, Rosado et al. 2019). Besides, some species have been reported as pathogens of animals and humans, saprobes and endophytes and been also reported as hyperparasites on other fungi (Sandoval-Denis et al. 2015, Sandoval-Denis et al. 2016, Zhou et al. 2016, Velázquez-Jiménez et al. 2019). Furthermore, some species have shown the ability to produce medicinal compounds or their potential as biological agents to control plant diseases (Köhl et al. 2015, Khan et al. 2016, Adorisio et al. 2019).

During the investigation of pathogenic fungi on leaf spots of medicinal plants in Yunnan Province, China, two new species *Cladosporiumyunnanensis* and *C.paris* were identified, based on morphology and multi-gene phylogenetic analysis. Full descriptions, illustrations and update of the phylogenetic backbone tree for *Cladosporium* are provided as well.

## Materials and methods

**Isolation and morphological examination**

Leaf specimens with disease symptoms of cultivated *Parispolyphylla* were collected from Dali, Yunnan Province, China in October and November 2020 and taken back to the laboratory in an envelope. The leaves were kept at 4°C in Zip-lock plastic bags before they were processed in the laboratory. Single spore isolations were made onto potato dextrose agar (PDA). After 8–10 hours, a single germinating conidia was transferred aseptically to a new PDA plate to obtain cultures and grow at 20–25°C in daylight (Chomnunti et al. 2014).

The cultures are deposited in Kunming Institute of Botany, Chinese Academy of Sciences (KUNCC) and China General Microbiological Culture Collection Center (CGMCC). Cultures are deposited at the Herbarium of Cryptogams Kunming Institute of Botany Academia Sinica (Herb. HKAS). Facesoffungi and Index Fungorum numbers were obtained as in Jayasiri et al. (2015) and Index Fungorum.

**DNA extraction, PCR ampliﬁcation and sequencing**

Genomic DNA was extracted from fresh mycelium grown on PDA at room temperature (25°C). The Trelief^TM^ Plant Genomic DNA Kit (TSP101) was used to extract DNA according to the manufacturer’s instructions. ITS, TEF1-α and ACT gene regions were amplified using the primer pairs ITS1/ITS4, EF1-728F/EF1-986R and ACT–512F/ACT–783R. The final volume of the PCR reaction was 25 µl and contained 12.5 µl of 2 × Power Taq PCR MasterMix (a premix and ready to use solution, including 0.1 Units/µl Taq DNA Polymerase, 500 µM dNTP Mixture each (dATP, dCTP, dGTP, dTTP), 20 mM Tris–HCl pH 8.3, 100 mM KCl, 3 mM MgCl_2_, stabiliser and enhancer), 1 μl of each primer (10 μM), 1 µl genomic DNA extract and 9.5 µl deionised water. The PCR thermal cycle programme for ITS, TEF1-α and ACT amplification was as follows: initial denaturation of 94°C for 3 minutes, followed by 35 cycles of denaturation at 94°C for 45 seconds, annealing at 56°C for 50 seconds, elongation at 72°C for 1 minute and the final extension at 72°C for 10 minutes. PCR products were purified using minicolumns, purification resin and buffer according to the manufacturer’s protocols (Amershamproduct code: 27–9602–01). The sequencing works were carried by Tsingke Biological Engineering Technology and Services Co., Ltd (Yunnan, P.R. China).

**Phylogenetic analysis**

Sequence data for relevant strains were downloaded from GenBank following latest publications (Freitas 2018, Iturrieta-González et al. 2021, Zimowska et al. 2021). The sequences aligned using MAFFTv.7 (http://mafft.cbrc.jp/alignment/server/) (Katoh and Standley 2013) and optimised manually when needed. The aligned dataset was analysed by Maximum Likelihood (ML) and Bayesian Inference (BI).

**Figure 1.**
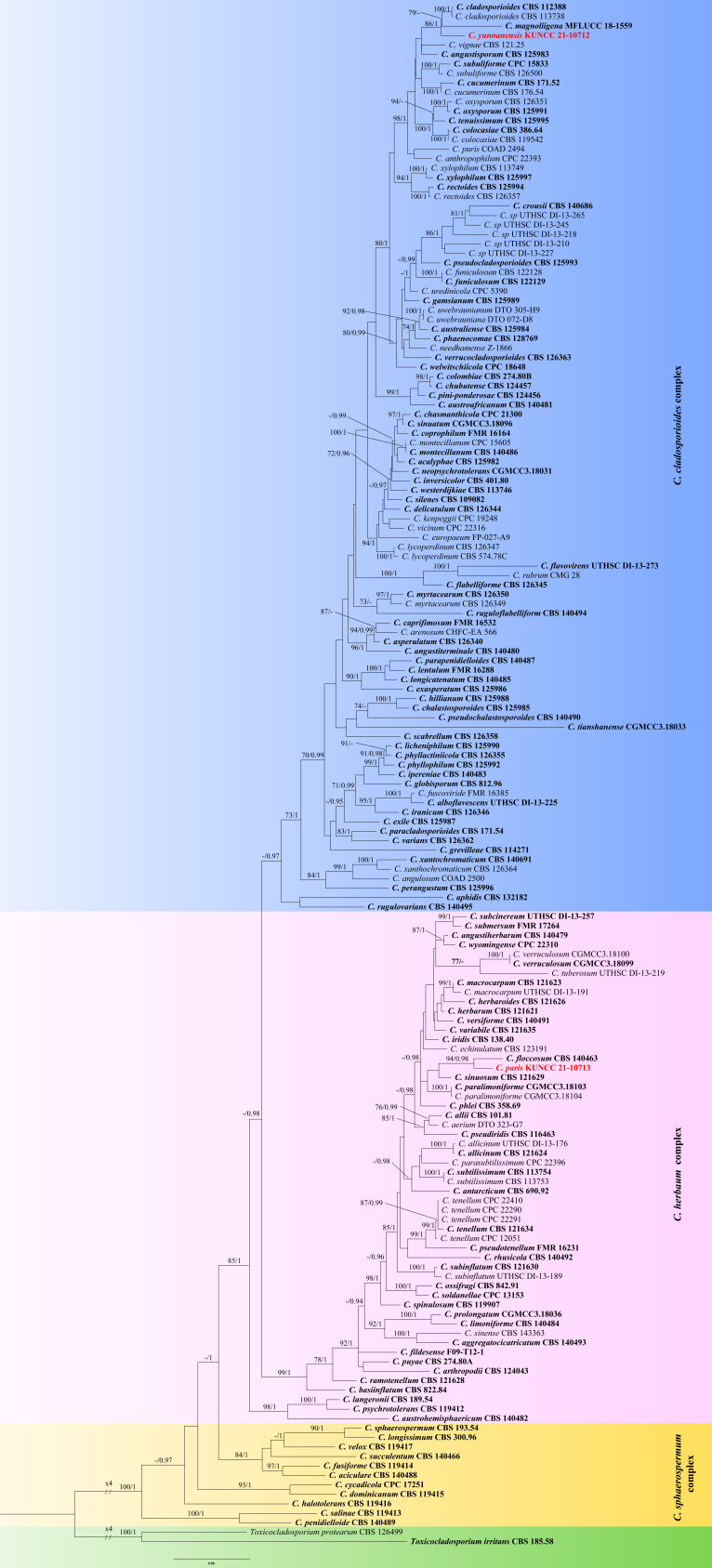
Maximum Likelihood (ML) tree obtained from the combined analysis of ITS, TEF1-α and ACT sequences of 161 strains from *Cladosporium*. The tree is rooted with *Toxicocladosporiumirritans* (CBS 185.58) and *T.protearum* (CBS 126499). Numbers on the branches represent ML bootstrap support values (MLBS) ≥70%, followed by Bayesian posterior probabilities (PP) ≥ 0.95, lower values are indicated as “-”. Names of species newly described are indicated in red and ex-type strains and reference specimens are indicated in bold. Branch lengths are proportional to distance.

Maximum Likelihood analysis was performed by using RAxMLGUI v.1.3 (Silvestro and Michalak 2012). The optimal ML tree search was conducted with 1,000 separate runs using the default algorithm of the programme from a random starting tree for each run. The final tree was selected amongst suboptimal trees from each run by comparing the likelihood scores using the GTR+GAMMA substitution model. Maximum Likelihood bootstrap values equal to or greater than 70% were given as the first set of numbers above the nodes in the resulting ML tree (Fig. 1).

Bayesian analysis was conducted with MrBayes v.3.1.2 (Ronquist and Huelsenbeck 2003) to evaluate posterior probabilities (Rannala and Yang 1996) by Markov Chain Monte Carlo sampling (MCMC). The best-fit models of evolution were estimated by MrModeltest v.2.2 (Nylander 2004). ITS selected the SYM+I+G model with inverse gamma-distributed rate in Bayesian analyses. TEF1-α and ACT selected the GTR+I+G model with inverse gamma-distributed rate in Bayesian analyses. The robustness of ML analyses was evaluated by bootstrap support (MLBS). The parameter settings, used in these analyses, were two simultaneous runs of 10,000,000 generations and four Markov chains, sampled every 1,000 generations. The 50% majority rule consensus tree and posterior probability values (PP) were calculated after discarding the first 25% of the samples. A PP value of ≥ 0.95 was considered significant (Hespanhol et al. 2019).

The phylogenetic trees were viewed and optimised in FigTree v.1.2.2 (Rambaut and Drummond 2008) and edited further using Microsoft Office PowerPoint. Newly-generated sequences in this study were deposited in GenBank (Table 1).

## Taxon treatments

### Cladosporium yunnanensis

H.W. Shen, Y.X. Xu, H.Y. Su & Z.L. Luo sp. nov.

C6DA32AB-EEE6-595C-98BA-4DA0C7F227ED

558843

Facesoffungi number: FoF 10538

#### Materials

- **Type status:** Holotype. **Occurrence:** recordedBy: Yue-Xin Xu; **Taxon:** scientificName: *Cladosporiumyunnanensis*; kingdom: Fungi; phylum: Ascomycota; class: Dothideomycetes; order: Capnodiales; family: Cladosporiaceae; genus: Cladosporium; **Location:** locationRemarks: China, Yunnan Province, Dali, on diseased leaves of *Parispolyphylla*, 2 October 2020; **Event:** day: 2020; habitat: leaf spots of *Parispolyphylla*; **Record Level:** collectionID: 1CL JD 5-1-4; collectionCode: Y-23

#### Description

**Figure 2.**
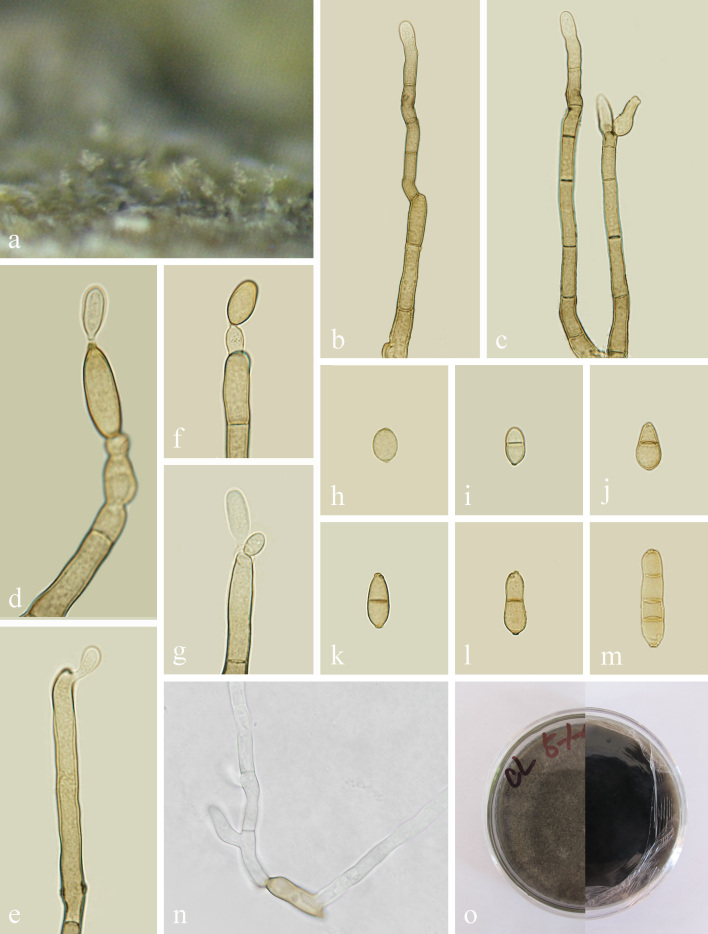
*Cladosporiumyunnanensis* **(KUN-HKAS 121704, holotype). a** Colonies; **b-c** Conidiophores; **d-g** Conidiogenous cells with conidia; **h-m** Conidia; **n** Germinating conidium; **o** Culture on PDA from above and reverse. Scale bars: **b-d** = 20 μm; **e-k** = 15 μm; **l** = 30 μm.

**Asexual morph**: hyphomycetous (Fig. 2). Mycelium superficial and immersed, composed of septate, branched, subhyaline, smooth-walled, 1–3 μm wide. Conidiophores macronematous, 127–190 × 4–6 μm (x̄ = 158.2 × 5.1 μm, n = 15), solitary or in small loose groups, erect to slightly flexuous, non-nodulose, sometimes subnodulose at the uppermost apex, unbranched, 0–6 septate, sometimes slightly constricted at septa, pale brown, smooth, sometimes somewhat irregularly rough-walled or verruculose. Conidiogenous cells terminal and intercalary, loci crowded at the apex forming clusters of pronounced scars, 1–2 conidiogenous loci formed at about the same level, loci often situated at lateral shoulders due to sympodial proliferation, loci 1–2 μm diam. Conidia solitary or in short unbranched chains, straight to slightly curved, cylindrical-oblong, 7–19 × 5–7 μm (x̄ = 13.2 × 5.7 μm, n = 30), 0–3 septate, sometimes slightly constricted at the septa, pale to pale medium olivaceous-brown. **Sexual morph**: Undetermined.

**Culture characteristics**: Colonies on PDA attaining 25 mm diam. after 7 d, 45 mm diam. after 14 d and covering the whole Petridish after 30 d, dark green to olive green, velvety, furrowed; reverse dark green to black.

**Material examined**: China, Yunnan Province, Dali, on diseased leaves of *Parispolyphylla*, 2 October 2020, Y.X. Xu, Y-23. (KUN-HKAS 121704, **holotype**), ex-type living culture CGMCC 3.20622 = KUNCC 21-10712

#### Etymology

“*yunnanensis*” refers to Yunnan Province, China, where the species was collected.

#### Distribution

China, Yunnan Province, Dali, on diseased leaves of *Parispolyphylla*

#### Notes

Based on the multi-locus phylogenetic analysis (Fig. 1), *Cladosporiumyunnanensis* grouped in a well-supported clade, together with *C.cladosporioides* and *C.magnoliigena*. However, the genetic distance allows it to be considered a distinct species within the clade (Fig. 1). Morphologically, *C.yunnanensis* has much shorter conidiophores than *C.cladosporioides* (up to 190 μm vs. up to 350 μm), but longer than *C.magnoliigena* (up to 190 μm vs. up to 150 μm). Moreover, the new species differs from *C.cladosporioides* by the smaller conidiogenous cells (7–19 × 5–7 μm vs. 4–18 × 2–5 μm), but larger than *C.magnoliigena* (7–19 × 5–7 μm vs. 4–18 × 2–5 μm) (Bensch et al. 2012, Jayasiri et al. 2019). The BLAST analysis of TEF1-α and ACT shows that *C.yunnanensis* (KUN-HKAS 121704) is different from *C.cladosporioides* (CBS 112388) by 16 and 10 nucleotide differences, respectively and the comparison of TEF1-α between *C.yunnanensis* (KUN-HKAS 121704) and *C.magnoliigena* (CBS 140463) reveals 33 nucleotide differences.

### Cladosporium paris

H.W. Shen, Y.X. Xu, H.Y. Su & Z.L. Luo sp. nov.

3C7F2601-1E71-567A-9D18-73083DAB684B

558844

Facesoffungi number: FoF 10539

#### Materials

- **Type status:** Holotype. **Occurrence:** recordedBy: Yue-Xin Xu; **Taxon:** scientificName: *Cladosporiumparis*; kingdom: Fungi; phylum: Ascomycota; class: Dothideomycetes; order: Capnodiales; family: Cladosporiaceae; genus: Cladosporium; **Location:** locationRemarks: China, Yunnan Province, Dali, on diseased leaves of *Parispolyphylla*; **Event:** year: 2020; habitat: leaf spots of *Parispolyphylla*; **Record Level:** collectionID: 2CL JD 18-2-1; collectionCode: Y-27

#### Description

**Figure 3.**
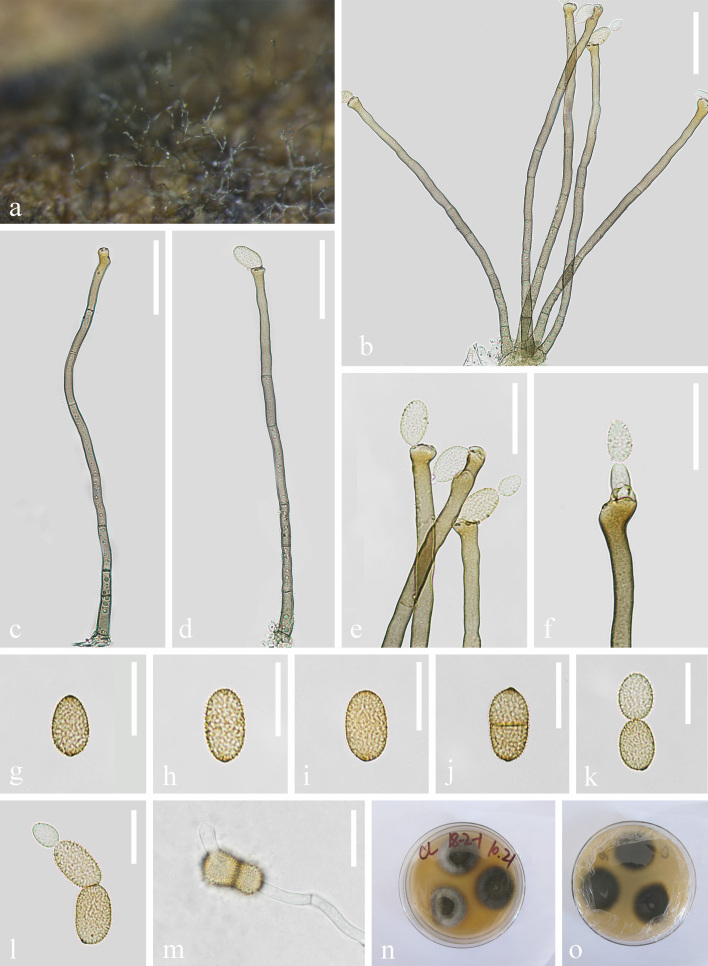
*Cladosporiumparis* **(KUN-HKAS 121701, holotype)**. **a** Colonies on leaves; **b,c** Conidiophores; **d** Conidiophore with conidium; **e,f** Conidiogenous cells with conidia; **g-l** Conidia; **m** Germinating conidium; **n,o** Culture on PDA from above and reverse. Scale bars: **b-d** = 50 μm; **e,f** = 30 μm; **g-m** = 20 μm.

**Asexual morph**: hyphomycetous (Fig. 3). Mycelium immersed and superficial, composed of septate, constricted at septa, unbranched, subhyaline, smooth hyphae, 2–6 μm wide. Conidiophores macronematous, 209–285 × 5–8 μm (x̄ = 246.9 × 6.5 μm, n = 15), solitary or in small fascicles, erect to slightly flexuous, sometimes slightly geniculate, non-nodulose, sometimes subnodulose at the uppermost apex, unbranched, 0–6 septate, sometimes slightly constricted at septa, pale to olivaceous-brown, smooth or almost so. Conidiogenous cells cylindrical, sometimes geniculate-sinuous, proliferation of sympodia with up to 5 conidiogenous loci, often crowded at the apex. Conidia 13–21 × 7–12 μm (x̄ = 17 × 9.7 μm, n = 30), solitary or catenate, usually in simple chains, broadly ellipsoid-ovoid, rather pale, pale olivaceous or olivaceous-brown, verruculose, ends usually broadly rounded. **Sexual morph**: Undetermined.

**Culture characteristics**: Colonies on PDA attaining 21 mm diam. after 7 d, 40 mm diam. after 14 d and covering the whole Petridish after 30 d, radially folded, furrowed, margin irregularly undulate; reverse olivaceous grey.

**Material examined**: China, Yunnan Province, Dali, on diseased leaves of *Parispolyphylla*, 16 October 2020, Y.X. Xu, Y-27. (KUN-HKAS 121701, **holotype**), ex-type living culture CGMCC 3.20623 = KUNCC 21-10713.

#### Etymology

“*paris*” refers to the host genus, *Paris*.

#### Distribution

China, Yunnan Province, Dali, on diseased leaves of *Parispolyphylla*

#### Notes

Phylogenetic analysis showed that *Cladosporiumparis* is closely related to *C.floccosum* (Fig. 1). Morphologically, our new isolate is distinguished from *C.floccosum* by its longer conidiophores (up to 285 μm vs. up to 100 μm) and larger conidiogenous cells (13–21 × 7–12 μm vs. 8–15 × 6–8.5 μm). In addition, conidia of *C.paris* are 0–3 septate, while *C.floccosum* are 0–1 septate (Sandoval-Denis et al. 2016). A comparison of the TEF1-α and ACT between *C.paris* (KUN-HKAS 121701) and *C.floccosum* (CBS 140463) reveals 3 and 16 nucleotide differences, respectively, which indicates that they are distinct taxa.

## Analysis

**Phylogenetic analysis**

The combined ITS, TEF1-α and ACT dataset consisted of 161 sequences representing all genera of the *Cladosporium* with *Toxicocladosporiumirritans* (CBS 185.58) and *T.protearum* (CBS 126499) as outgroup taxa. The best scoring RaxML tree with the final ML optimisation likelihood value of -24601.202740 is shown here (Fig. 1). The alignment comprised 1297 characters including gaps. The matrix had 775 distinct alignment patterns, with 15.38% undetermined characters or gaps. Estimated base frequencies were as follows: A = 0.228337, C = 0.293636, G = 0.250484, T = 0.227544; substitution rates AC = 1.726214, AG = 3.618770, AT = 1.752951, CG = 1.098108, CT = 5.802327, GT = 1.000000; Tree-Length = 7.357731.

Phylogenetic analyses of combined ITS, TEF1-α and ACT sequence data showed that the two new isolates of *Cladosporiumyunnanensis* (KUN-HKAS 121704) and *C.paris* (KUN-HKAS 121701) grouped with members of *Cladosporium*. *Cladosporiumyunnanensis* (KUN-HKAS 121704) clustered with *C.cladosporiordes* (CBS 112388 and CBS 113738) and *C.magnoliigena* (MFLUCC 18-1559), but in an independent lineage with significant bootstrap (86 ML/1.00 PP). *Cladosporiumparis* (KUN-HKAS 121701) formed a distinct lineage and sister to *C.floccosum* (CBS 140463) and basal to the genus with highly-supported value (94 ML/0.98 PP).

## Discussion

In our study, based on the typical morphological features (Schubert et al. 2007, Zalar et al. 2007, Dugan et al. 2008, Bensch et al. 2010), *Cladosporiumyunnanensis* and *C.paris* belong to the *C.cladosporioides* and *C.herbarum* species complex, respectively. The ITS sequences of the two new species are identical under the common barcode for fungi as previously reported studies for many other *Cladosporium* species (Bensch et al. 2010, Bensch et al. 2012, Marin-Felix et al. 2017). Therefore, multi-gene phylogenetic analysis (ITS, TEF1-α and ACT) can further prove the taxonomy of the two species in *Cladosporium*, which is consistent with the result by morphological features.

*Cladosporium* species are found as the dominant fungal genera in indoor and outdoor environments and are also important as saprobes and endophytes which have been screened from grains, fruits and chilled meat (Fradkin et al. 1987, Bullerman 2003, Hassan et al. 2021). However, *Cladosporiumyunnanensis* and *C.paris* have been isolated from leaves of *Parispolyphylla* in Yunnan Province, China for the first time. Studies indicate that investigation on new hosts for fungi diversity would lead to the discovery of new fungal species and expand species resources (Hyde et al. 2018, Hyde et al. 2020). Certain *Cladosporium* species have been reported as producers of mycotoxin and to cause fungal allergies, particularly rhinitis and asthma. (Horner et al. 1995, Kurup 2003, Matheson et al. 2005, Simon-Nobbe et al. 2008, Mercier et al. 2013, Alwatban et al. 2014 , Segers et al. 2015). Both new species are isolated from diseased spots on plant leaves and many species of this genus are reported as plant pathogens, so they also have the potential to cause plant diseases. To determine whether these fungi are plant pathogens or have long-term adverse reactions on human health, pathogenicity determination and secondary metabolites of *Cladosporium* can be the focus of our future research.

## Supplementary Material

XML Treatment for Cladosporium yunnanensis

XML Treatment for Cladosporium paris

## Figures and Tables

**Table 1.** Isolates and sequences used in this study (newly-generated sequences are indicated with a “*”, strains isolated from the holotype and reference specimens are indicated in bold).

**Species**	**Strain number**	**GenBank Accession Numbers**		
		**ITS**	**TEF1-α**	**ACT**
***Cladosporiumacalyphae***	**CBS 125982**	**NR_119835**	**HM148235**	**HM148481**
***C.aciculare***	**CBS 140488**	**KT600411**	**KT600509**	**KT600607**
*C.aerium*	DTO 323-G7	MF472899	MF473326	MF473749
***C.aggregatocicatricatum***	**CBS 140493**	**NR_152300**	**KT600547**	**KT600645**
***C.alboflavescens***	**UTHSC DI-13-225**	**LN834420**	**LN834516**	**LN834604**
***C.allicinum***	**CBS 121624**	**NR_152266**	**EF679425**	**EF679502**
*C.allicinum*	UTHSC DI-13-176	LN834354	LN834450	LN834538
***C.allii***	**CBS 101.81**	**JN906977**	**JN906983**	**JN906996**
*C.angulosum*	COAD 2500	MK253346	MK293786	MK249989
***C.angustiherbarum***	**CBS 140479**	**NR_152286**	**KT600475**	**KT600574**
***C.angustisporum***	**CBS 125983**	**NR_111530**	**HM148236**	**HM148482**
***C.angustiterminale***	**CBS 140480**	**NR_152287**	**KT600476**	**KT600575**
***C.antarcticum***	**CBS 690.92**	**NR_121332**	**EF679405**	**EF679484**
*C.anthropophilum*	CPC 22393	MF472922	MF473349	MF473772
***C.aphidis***	**CBS 132182**	**JN906978**	**JN906984**	**JN906997**
*C.arenosum*	CHFC-EA 566	MN879328	MN890011	MN890008
***C.arthropodii***	**CBS 124043**	**NR_120011**	**JN906985**	**JN906998**
***C.asperulatum***	**CBS 126340**	**NR_119836**	**HM148239**	**HM148485**
***C.australiense***	**CBS 125984**	**NR_119837**	**HM148240**	**HM148486**
***C.austroafricanum***	**CBS 140481**	**NR_152288**	**KT600478**	**KT600577**
***C.austrohemisphaericum***	**CBS 140482**	**KT600382**	**KT600479**	**KT600578**
***C.basiinflatum***	**CBS 822.84**	**NR_111531**	**HM148241**	**HM148487**
***C.caprifimosum***	**FMR 16532**	**LR813198**	**LR813210**	**LR813205**
***C.chalastosporoides***	**CBS 125985**	**NR_119838**	**HM148242**	**HM148488**
***C.chasmanthicola***	**CPC 21300**	**NR_152307**	**KY646227**	**KY646224**
***C.chubutense***	**CBS 124457**	**NR_119728**	**FJ936161**	**FJ936165**
***C.cladosporioides***	**CBS 112388**	**NR_119839**	**HM148244**	**HM148490**
*C.cladosporioides*	CBS 113738	HM148004	HM148245	HM148491
***C.colocasiae***	**CBS 386.64**	**NR_119840**	**HM148310**	**HM148555**
*C.colocasiae*	CBS 119542	HM148066	HM148309	HM148554
***C.colombiae***	**CBS 274.80B**	**NR_119729**	**FJ936163**	**FJ936166**
***C.coprophilum***	**FMR 16164**	**LR813201**	**LR813213**	**LR813207**
***C.crousii***	**CBS 140686**	**LN834431**	**LN834527**	**LN834615**
***C.cucumerinum***	**CBS 171.52**	**NR_119841**	**HM148316**	**HM148561**
*C.cucumerinum*	CBS 176.54	HM148078	HM148322	HM148567
***C.cycadicola***	**CPC 17251**	**KJ869122**	**KJ869236**	**KJ869227**
***C.delicatulum***	**CBS 126344**	**MH863920**	**HM148325**	**HM148570**
***C.dominicanum***	**CBS 119415**	**DQ780353**	**JN906986**	**EF101368**
*C.echinulatum*	CBS 123191	JN906980	JN906987	JN906999
*C.europaeum*	FP-027-A9	MH102078	MH102121	MH102068
***C.exasperatum***	**CBS 125986**	**NR_119843**	**HM148334**	**HM148579**
***C.exile***	**CBS 125987**	**NR_111532**	**HM148335**	**HM148580**
***C.fildesense***	**F09-T12-1**	**JX845290**	**MN233633**	**MN233632**
***C.flabelliforme***	**CBS 126345**	**NR_119844**	**HM148336**	**HM148581**
***C.flavovirens***	**UTHSC DI-13-273**	**LN834440**	**LN834536**	**LN834624**
***C.floccosum***	**CBS 140463**	**LN834416**	**LN834512**	**LN834600**
***C.funiculosum***	**CBS 122129**	**NR_119845**	**HM148338**	**HM148583**
*C.funiculosum*	CBS 122128	HM148093	HM148337	HM148582
*C.fuscoviride*	FMR 16385	LR813200	LR813212	LR813206
***C.fusiforme***	**CBS 119414**	**DQ780388**	**JN906988**	**EF101372**
***C.gamsianum***	**CBS 125989**	**NR_111533**	**HM148339**	**HM148584**
***C.globisporum***	**CBS 812.96**	**NR_111534**	**HM148340**	**HM148585**
***C.grevilleae***	**CBS 114271**	**NR_119960**	**JF770472**	**JF770473**
***C.halotolerans***	**CBS 119416**	**DQ780364**	**JN906989**	**EF101397**
***C.herbaroides***	**CBS 121626**	**NR_119655**	**EF679432**	**EF679509**
***C.herbarum***	**CBS 121621**	**NR_119656**	**EF679440**	**EF679516**
***C.hillianum***	**CBS 125988**	**NR_119846**	**HM148341**	**HM148586**
***C.inversicolor***	**CBS 401.80**	**NR_111535**	**HM148345**	**HM148590**
***C.ipereniae***	**CBS 140483**	**NR_152290**	**KT600491**	**KT600589**
***C.iranicum***	**CBS 126346**	**NR_111536**	**HM148354**	**HM148599**
***C.iridis***	**CBS 138.40**	**NR_111271**	**EF679447**	**EF679523**
*C.kenpeggii*	CPC 19248	KY646222	KY646228	KY646225
***C.langeronii***	**CBS 189.54**	**DQ780379**	**JN906990**	**EF101357**
***C.lentulum***	**FMR 16288**	**LR813203**	**LR813215**	**LR813209**
***C.licheniphilum***	**CBS 125990**	**NR_119847**	**HM148355**	**HM148600**
***C.limoniforme***	**CBS 140484**	**KT600397**	**KT600494**	**KT600592**
***C.longicatenatum***	**CBS 140485**	**NR_152291**	**KT600500**	**KT600598**
***C.longissimum***	**CBS 300.96**	**DQ780352**	**EU570259**	**EF101385**
*C.lycoperdinum*	CBS 126347	MH863923	HM148356	HM148601
*C.lycoperdinum*	CBS 574.78C	HM148115	HM148359	HM148604
***C.macrocarpum***	**CBS 121623**	**NR_119657**	**EF679453**	**EF679529**
*C.macrocarpum*	UTHSC DI-13-191	LN834379	LN834475	LN834563
***C.magnoliigena***	**MFLUCC 18-1559**	**MK347813**	**MK340864**	-
***C.montecillanum***	**CBS 140486**	**NR_152292**	**KT600504**	**KT600602**
*C.montecillanum*	CPC 15605	KT600407	KT600505	KT600603
*C.myrtacearum*	CBS 126349	MH863925	HM148360	HM148605
***C.myrtacearum***	**CBS 126350**	**NR_119849**	**HM148361**	**HM148606**
*C.needhamense*	Z-1866	MF473142	MF473570	MF473991
***C.neopsychrotolerans***	**CGMCC3.18031**	**KX938383**	**KX938400**	**KX938366**
***C.ossifragi***	**CBS 842.91**	**NR_121333**	**EF679459**	**EF679535**
***C.oxysporum***	**CBS 125991**	**NR_152267**	**HM148362**	**HM148607**
*C.oxysporum*	CBS 126351	MH863927	HM148363	HM148608
***C.paracladosporioides***	**CBS 171.54**	**NR_119850**	**HM148364**	**HM148609**
***C.paralimoniforme***	**CGMCC3.18103**	**KX938392**	**KX938409**	**KX938375**
*C.paralimoniforme*	CGMCC3.18104	KX938393	KX938410	KX938376
***C.parapenidielloides***	**CBS 140487**	**NR_152293**	**KT600508**	**KT600606**
*C.parasubtilissimum*	CPC 22396	MF473171	MF473594	MF474019
*C.paris* sp. nov.*	KUN HKAS 121701*	OK338503*	OL825681*	OL466938*
***C.penidielloide***	**CBS 140489**	**KT600412**	**KT600510**	**KT600608**
***C.perangustum***	**CBS 125996**	**NR_119851**	**HM148365**	**HM148610**
***C.phaenocomae***	**CBS 128769**	**NR_119950**	**JF499875**	**JF499881**
***C.phlei***	**CBS 358.69**	**NR_120013**	**JN906991**	**JN907000**
***C.phyllactiniicola***	**CBS 126355**	**NR_111537**	**HM148397**	**HM148642**
***C.phyllophilum***	**CBS 125992**	**NR_111538**	**HM148398**	**HM148643**
***C.pini-ponderosae***	**CBS 124456**	**NR_119730**	**FJ936164**	**FJ936167**
***C.prolongatum***	**CGMCC3.18036**	**KX938394**	**KX938411**	**KX938377**
***C.pseudiridis***	**CBS 116463**	**NR_111272**	**EF679461**	**EF679537**
***C.pseudochalastosporoides***	**CBS 140490**	**NR_152296**	**KT600513**	**KT600611**
***C.pseudocladosporioides***	**CBS 125993**	**NR_119852**	**HM148402**	**HM148647**
***C.pseudotenellum***	**FMR 16231**	**LR813145**	**LR813196**	**LR813146**
***C.psychrotolerans***	**CBS 119412**	**DQ780386**	**JN906992**	**EF101365**
*C.puris*	COAD 2494	MK253338	MK293778	MK249981
***C.puyae***	**CBS 274.80A**	**NR_152298**	**KT600516**	**KT600614**
***C.ramotenellum***	**CBS 121628**	**NR_119658**	**EF679462**	**EF679538**
***C.rectoides***	**CBS 125994**	**NR_111539**	**HM148438**	**HM148683**
*C.rectoides*	CBS 126357	MH863933	HM148439	HM148684
***C.rhusicola***	**CBS 140492**	**NR_152299**	**KT600539**	**KT600637**
*C.rubrum*	CMG 28	MN053018	MN066644	MN066639
***C.ruguloflabelliform***	**CBS 140494**	**KT600458**	**KT600557**	**KT600655**
***C.rugulovarians***	**CBS 140495**	**KT600459**	**KT600558**	**KT600656**
***C.salinae***	**CBS 119413**	**DQ780374**	**JN906993**	**EF101390**
***C.scabrellum***	**CBS 126358**	**NR_119853**	**HM148440**	**HM148685**
***C.silenes***	**CBS 109082**	**NR_111270**	**EF679429**	**EF679506**
*C.sinense*	CBS 143363	MF473252	MF473675	MF474102
***C.sinuatum***	**CGMCC3.18096**	**KX938385**	**KX938402**	**KX938368**
***C.sinuosum***	**CBS 121629**	**NR_119659**	**EF679464**	**EF679540**
***C.soldanellae***	**CPC 13153**	**NR_120014**	**JN906994**	**JN907001**
*C.* sp.	UTHSC DI-13-227	LN834422	LN834518	LN834606
*C.* sp.	UTHSC DI-13-245	LN834429	LN834525	LN834613
*C.* sp.	UTHSC DI-13-265	LN834435	LN834531	LN834619
*C.* sp.	UTHSC DI-13-218	LN834418	LN834514	LN834602
*C.* sp.	UTHSC DI-13-210	LN834414	LN834510	LN834598
***C.sphaerospermum***	**CBS 193.54**	**NR_111222**	**EU570261**	**EU570269**
***C.spinulosum***	**CBS 119907**	**NR_119660**	**EF679466**	**EF679542**
***C.subcinereum***	**UTHSC DI-13-257**	**NR_148193**	**LN834529**	**LN834617**
*C.subinflatum*	UTHSC DI-13-189	LN834391	LN834487	LN834575
***C.subinflatum***	**CBS 121630**	**NR_119661**	**EF679467**	**EF679543**
***C.submersum***	**FMR 17264**	**LR813144**	**LR813197**	**LR813195**
***C.subtilissimum***	**CBS 113754**	**NR_111273**	**EF679475**	**EF679551**
*C.subtilissimum*	CBS 113753	EF679396	EF679474	EF679550
***C.subuliforme***	**CBS 126500**	**NR_119854**	**HM148441**	**HM148686**
*C.subuliforme*	CPC 15833	KT600453	KT600552	KT600650
***C.succulentum***	**CBS 140466**	**LN834434**	**LN834530**	**LN834618**
***C.tenellum***	**CBS 121634**	**NR_119662**	**EF679479**	**EF679555**
*C.tenellum*	CPC 22410	MF473280	MF473703	MF474130
*C.tenellum*	CPC 12051	EF679400	EF679478	EF679554
*C.tenellum*	CPC 22291	MF473279	MF473702	MF474129
*C.tenellum*	CPC 22290	MF473278	MF473701	MF474128
***C.tenuissimum***	**CBS 125995**	**NR_119855**	**HM148442**	**HM148687**
***C.tianshanense***	**CGMCC3.18033**	**KX938381**	**KX938398**	**KX938364**
*C.tuberosum*	UTHSC DI-13-219	LN834419	LN834515	LN834603
*C.uredinicola*	CPC 5390	AY251071	HM148467	HM148712
*C.uwebrauniana*	DTO 072-D8	MF473306	MF473729	MF474156
*C.uwebraunianum*	DTO 305-H9	MF473307	MF473730	MF474157
***C.variabile***	**CBS 121635**	**NR_119663**	**EF679481**	**EF679557**
***C.varians***	**CBS 126362**	**NR_119856**	**HM148470**	**HM148715**
***C.velox***	**CBS 119417**	**DQ780361**	**JN906995**	**EF101388**
***C.verrucocladosporioides***	**CBS 126363**	**NR_111540**	**HM148472**	**HM148717**
***C.verruculosum***	**CGMCC3.18099**	**KX938388**	**KX938405**	**KX938371**
*C.verruculosum*	CGMCC3.18100	KX938389	KX938406	KX938372
***C.versiforme***	**CBS 140491**	**NR_152297**	**KT600515**	**KT600613**
*C.vicinum*	CPC 22316	MF473311	MF473734	MF474161
*C.vignae*	CBS 121.25	HM148227	HM148473	HM148718
***C.welwitschiicola***	**CPC 18648**	**NR_152308**	**KY646229**	**KY646226**
***C.westerdijkiae***	**CBS 113746**	**HM148061**	**HM148303**	**HM148548**
***C.wyomingense***	**CPC 22310**	**MF473315**	**MF473738**	**MF474165**
*C.xanthochromaticum*	CBS 126364	HM148122	HM148366	HM148611
***C.xantochromaticum***	**CBS 140691**	**LN834415**	**LN834511**	**LN834599**
***C.xylophilum***	**CBS 125997**	**NR_111541**	**HM148476**	**HM148721**
*C.xylophilum*	CBS 113749	HM148228	HM148474	HM148719
*C.yunnanensis* sp. nov.*	KUN HKAS 121704*	OK338502*	OL825680*	OL466937*
***Toxicocladosporiumirritans***	**CBS 185.58**	**NR_152316**	-	**LT821375**
***Toxicocladosporiumprotearum***	**CBS 126499**	**NR_152321**	-	**LT821379**
